# Order without design

**DOI:** 10.1186/1742-4682-7-12

**Published:** 2010-04-14

**Authors:** Alexei Kurakin

**Affiliations:** 1 Department of Pathology, Beth Israel Deaconess Medical Center and Harvard Medical School, Boston, MA 02215, USA

## Abstract

Experimental reality in molecular and cell biology, as revealed by advanced research technologies and methods, is manifestly inconsistent with the design perspective on the cell, thus creating an apparent paradox: where do order and reproducibility in living systems come from if not from design?

I suggest that the very idea of biological design (whether evolutionary or intelligent) is a misconception rooted in the time-honored and thus understandably precious error of interpreting living systems/organizations in terms of classical mechanics and equilibrium thermodynamics. This error, introduced by the founders and perpetuated due to institutionalization of science, is responsible for the majority of inconsistencies, contradictions, and absurdities plaguing modern sciences, including one of the most startling paradoxes - although almost everyone agrees that any living organization is an open nonequilibrium system of continuous energy/matter flow, almost everyone interprets and models living systems/organizations in terms of classical mechanics, equilibrium thermodynamics, and engineering, i.e., in terms and concepts that are fundamentally incompatible with the physics of life.

The reinterpretation of biomolecules, cells, organisms, ecosystems, and societies in terms of open nonequilibrium organizations of energy/matter flow suggests that, in the domain of life, order and reproducibility do not come from design. Instead, they are natural and inevitable outcomes of self-organizing activities of evolutionary successful, and thus persistent, organizations co-evolving on multiple spatiotemporal scales as biomolecules, cells, organisms, ecosystems, and societies. The process of self-organization on all scales is driven by economic competition, obeys empirical laws of nonequilibrium thermodynamics, and is facilitated and, thus, accelerated by memories of living experience persisting in the form of evolutionary successful living organizations and their constituents.

## 

The cell is a fundamental building block of all living organisms. A typical cell represents a highly concentrated (300-400 mg/ml of proteins and RNA alone [1]) aqueous solution of macromolecules, small molecules, and ions enveloped in a semi-permeable lipid membrane. Due to their physicochemical and structural versatility, proteins perform the vast majority of biological functions in the cell. Individual proteins and self-assembled multi-protein complexes operate as exquisite nanomachines optimized by evolution for efficient performance of an immense variety of specific tasks required for proper functioning of the cell as a whole [2]. Through the regulatory circuitry of signal transduction pathways, various cellular programs control and direct cellular machinery in accordance with the evolutionary design of the cell.

To understand design means to control the designed. Naturally, in order to gain some measure of control over our diseases and inevitable aging and death, we direct our best efforts and resources at deciphering the evolutionary design of the cell, using our most reliable and successful analytical methods, reductionism and reverse engineering. The incessant and rapid progress of research technology enables us to isolate and analyze increasingly smaller parts on an increasingly larger scale with increasingly better precision and at an increasingly faster pace. We have sequenced our own genome and know the blueprints of almost every protein and RNA produced in our cells. Modern biophysical and computational methods allow us to analyze individual proteins on both population and single-molecule levels, as well as to scrutinize and model protein structure and dynamics at atomic resolution and on multiple timescales, from femtoseconds to seconds. Internationally coordinated initiatives in structural genomics aim at high-throughput determination of all relevant biomolecular structures, with thousands of protein structures being deposited in the Protein Data Bank each year. Mass spectrometry-based proteomics technologies allow us to obtain "parts lists" for virtually any macromolecular complex, organelle, or sub-cellular structure. Robotics-assisted large-scale protein interaction studies reveal the relationships between individual parts of the cellular machinery on the scale of whole proteomes. Hundreds of years' expertise in engineering is combined with the unprecedented powers of modern computational technologies to equip systems biology for the analysis and modeling of systems of almost any degree of complexity. Paradoxically, extraordinary advances in our understanding of the parts do not seem to bring about significant progress in our understanding of the whole. In fact, it appears that the design of the cell becomes increasingly elusive as experimental data accumulate.

Comparative analysis of the "parts lists" obtained for various organelles, sub-cellular structures, and macromolecular complexes suggests, for example, that there may be no pre-defined locations for individual proteins inside the cell, but rather statistically preferred ones. Moreover, even statistically preferred spatiotemporal distributions of proteins inside the cell are not fixed. Instead, they appear to change on multiple scales of space and time upon changes in the internal state of the cell and/or its environment [3,4]. The findings of large-scale protein interaction studies echo the results of proteomics studies, showing that many proteins have multiple interacting partners dispersed among diverse cellular locations and functionally distinct macromolecular complexes [5,6]. Quantitative visualization of fluorescently tagged proteins inside living cells shows that most, perhaps all, sub-cellular structures and macromolecular complexes exist not as pre-assembled and relatively stable structures, but as highly dynamic steady-state macromolecular organizations, conceptually similar to a treadmilling actin filament but of greater complexity. Steady-state sub-cellular structures and macromolecular complexes are sustained by the flow of energy and matter passing through them in the form of their resident components continuously entering and leaving macromolecular organizations with widely varying recruitment probabilities, residence times, and turnover rates [7,8]. Apparently, sub-cellular organization as a whole is in perpetual and rapid flux, with proteins and other molecules dynamically and incessantly partitioning and repartitioning on multiple timescales among spatially and functionally distinct cellular locations, complexes, and structures. Fittingly, studies focused on the elucidation of biological functions of individual proteins by traditional approaches show that many proteins can and do perform multiple and often unrelated functions inside the cell [9,10]. The multifunctionality of proteins, first recognized as a curiosity under the cliché "moonlighting proteins," is increasingly perceived today as an inherent property of proteins that can be assumed for most, perhaps all, of them. Altogether, the recent findings and discoveries amassed in various research fields with the help of advanced technologies and methods appear to converge on one and the same conclusion, suggesting that the localization, interactions, and functions of proteins in the cell are inherently ambiguous, i.e., unspecified. Needless to say, such a conclusion not only makes the inference of cellular design an increasingly elusive goal, it questions the very existence of such a thing as biological design. Yet, this seemingly radical conclusion is in perfect harmony with recent breakthroughs in our understanding of protein structure and dynamics.

Defying traditional, static views on proteins embodied in such metaphors as "locks," "keys," and "Lego blocks," recent technological and conceptual advances in protein biophysics have brought about a new image of the protein as a highly dynamic, versatile, and continuously changing physicochemical organization [11-13]. It is now evident that any protein exists in solution not as a pre-defined structure, but as a population of conformers incessantly interconverting on multiple timescales. A protein molecule continuously and stochastically samples its different conformations, undergoing relatively slow structural transitions between different families of related conformers and relatively fast transitions within a given conformer family [12,14]. The rates of interconversion are defined by the heights of energy barriers separating individual conformational states that are represented as relative energy minima in the protein energy landscape (Figure 1). Moreover, the energy landscape is not fixed. Binding of ligands, post-translational modifications, temperature, pressure, solvent and other factors may alter the shape of the energy landscape, triggering a redistribution of conformers and/or changing heights of the energy barriers separating alternative conformations [12,13,15]. Because different conformers can potentially bind different ligands and perform different cellular functions, inherent ambiguity in protein interactions, localization, and function is an inevitable and natural consequence of the conformational heterogeneity and structural plasticity of proteins [14,16].

**Figure 1:**
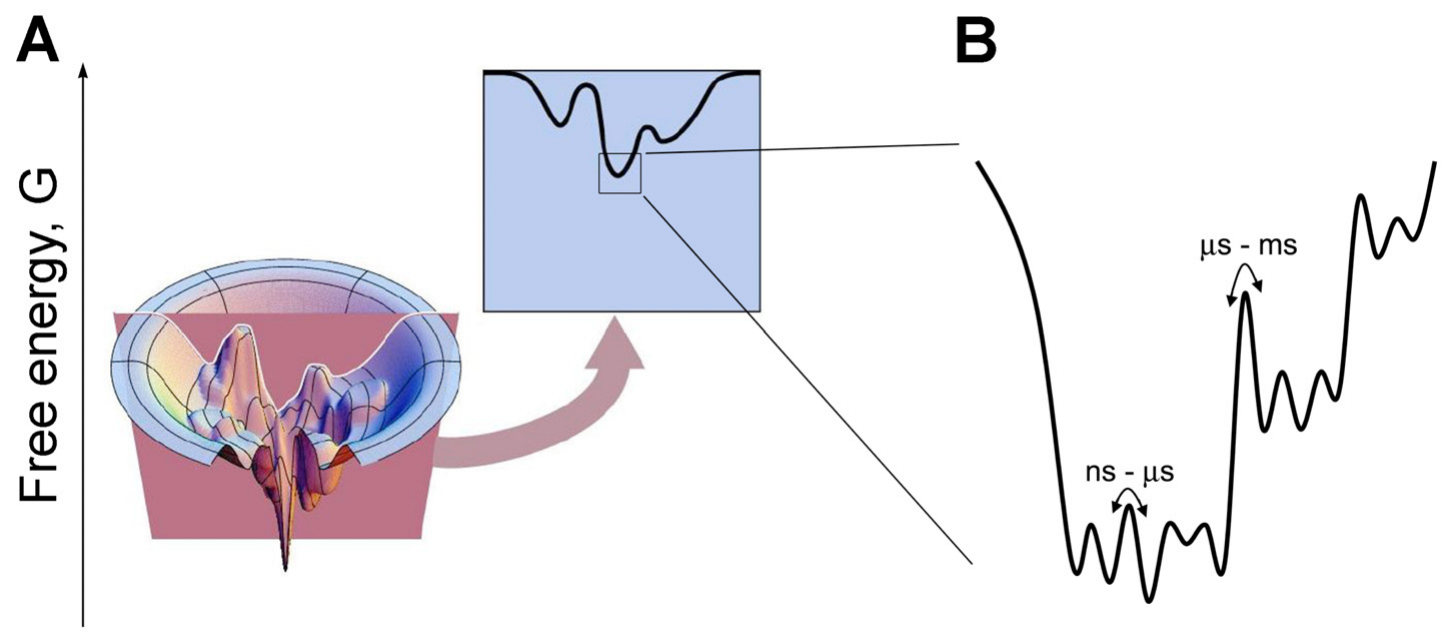
**The concept of the energy landscape**. **A**) As a ball rolling down a rugged landscape under the force of gravity strives to minimize its potential energy, a folding protein structure descending a virtual energy landscape strives to minimize a thermodynamic potential called the Gibbs free energy. The "real" energy landscape of a protein is highly multidimensional; however, many qualitative properties of the protein folding process such as, for example, multiplicity of folding pathways and intermediate energy minima or "traps" in which a partially folded structure may become stuck on its way to the bottom of the landscape are conveniently captured and visualized in low-dimensional sections of the energy landscape, as shown here. (Reprinted with permission from Ken Dill, http://www.dillgroup.ucsf.edu/). **B**) The bottom of the energy landscape, which corresponds to a native (folded) structure, is a rugged landscape in itself, meaning that any native protein structure exists in solution as a population of interconverting conformational states that are separated by energy barriers of varying heights. The latter define the probabilities and thus rates of interconversions. Interconversions on timescales of microseconds and slower usually correspond to large-scale collective (domain) motions within the protein structure, which are relatively rare. Loop motions and side-chain rotations typically occur on timescales of pico- to microseconds, while atom fluctuations occur on timescales of picoseconds and faster.

The so-called natively unfolded proteins, which do not possess a defined structure when isolated in solution, but acquire a structure upon interactions with other molecules, represent an extreme example of protein ambiguity and plasticity. The discovery of intrinsically disordered proteins came as a total surprise, for the concept of natively unfolded proteins is incommensurate within the design perspective on the cell [17]. Subsequent bioinformatics analyses of various genomes revealed that intrinsically disordered proteins are present in all domains of life, with the relative abundance of intrinsically disordered proteins in genomes rapidly increasing from archaea and eubacteria through single cell eukaryotes and multicellular eukaryotes. It is estimated that in mammals approximately 75% of signaling proteins and about 50% of all proteins contain at least one large unstructured region (> 30 amino acids), while about 25% of all proteins are fully disordered [18]. Since unstructured regions and proteins are apparently important for cellular functions [19], and structured regions and proteins are multi-conformational entities, the ordering and functioning of proteins inside the cell cannot possibly rely on the specificity provided by protein structure alone, but should be driven by some unknown principles that are different from, but complementary to, the conventional principles of molecular recognition embodied in the "lock-and-key" and "Lego block" metaphors. Structurally ambiguous and simply flexible proteins have *a choice*, for they can interact with different partners, join different macromolecular organizations, perform different actions, and contribute in different ways to the functioning of diverse macromolecular complexes and structures.

To summarize, the experimental reality in molecular and cell biology, as revealed by advanced technologies and methods, is manifestly inconsistent with the design perspective on the cell, creating an apparent paradox: where do order and reproducibility in living systems come from if not from design?

A recent theoretical study may provide an answer to this and other questions accumulated in biology over many years of research. This study demonstrates that the experimental reality in molecular and cell biology becomes largely devoid of paradoxes, inconsistencies and contradictions, and is thus best understood if the cell and biological organization in general are reinterpreted within an alternative paradigm of biological organization based on the concepts and empirical laws of nonequilibrium thermodynamics [20].

Whether explicitly stated or tacitly implied, the phenomena studied in molecular and cell biology are traditionally interpreted and rationalized within the conceptual framework of classical physics, i.e., classical mechanics and equilibrium thermodynamics. This is not particularly surprising, for the foundations of molecular and cell biology were laid down by physicists and biochemists whose mental structures and, thus, habitual interpretations were shaped by their rigorous training in classical physics and engineering. Accordingly, as exemplified by the first paragraph of this article, the conventional image of the cell came to carry within it all the familiar logic, inferences, and assumptions of classical physics and engineering, including the belief that the order and reproducibility observed in living systems are a consequence of design. What is really surprising is that, even though today very few scientists would argue that the cell is not an open *nonequilibrium *physicochemical system of interacting molecules, the vast majority of researchers working in molecular and cell biology continue to treat and interpret the cell and its components in terms of classical mechanics, equilibrium thermodynamics, and engineering, i.e., in terms and concepts that are fundamentally incompatible with the physical nature of the cell, thus faithfully reproducing and reinforcing the mistakes and misconceptions of the founders. This is in itself a good example demonstrating that many things in life can be faithfully reproduced in the absence of any design.

Studies of relatively simple inorganic nonequilibrium systems such as the Belousov-Zhabotinsky (BZ) reaction, Benard instability (Figure 2), and others show that creating a gradient (e.g., temperature, concentration, chemical) within a molecular system of interacting components normally causes a flux of energy/matter in the system and, as a consequence, the emergence of a countervailing gradient, which, in turn, may cause the emergence of another flux and another gradient, and so on. The resulting complex system of conjugated fluxes and coupled gradients is manifested as a spatiotemporal macroscopic order spontaneously emerging in an initially featureless, disordered system, provided the system is driven far enough away from equilibrium [21-23]. As a rule, the emergence of macroscopic order is a highly nonlinear, cooperative process. When a critical threshold value of flow rate is exceeded, the system spontaneously organizes itself by partitioning its components into interdependent and interconnected steady state macroscopic structures. The macrostructures emerging in far-from-equilibrium systems are of a steady-state nature in the sense that what is actually preserved and evolves over relevant timescales is an *organization *of relationships between interacting components, a form, but not physical components comprising a given macrostructure. Members come and go, but the organization persists. Normally, the same set of interacting microcomponents can generate multiple alternative organizational configurations differing in the organization of energy/matter exchanges transiently maintained among the interacting components that make up and flow through a given configuration. As a consequence, macrostructures emerging in far-from-equilibrium systems are dynamic in two different senses, for they display both configurational dynamics and flow dynamics [20].

**Figure 2:**
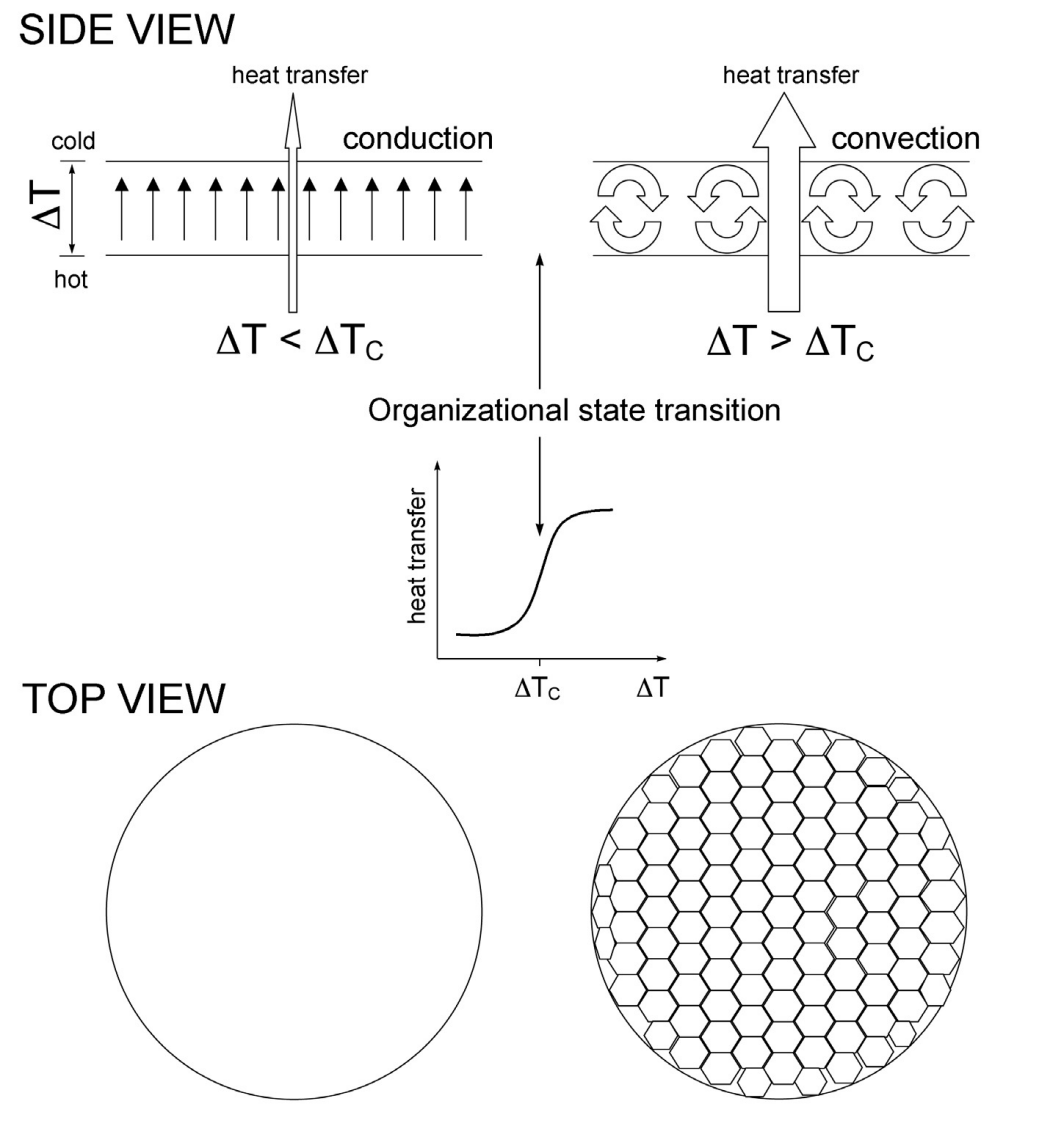
**The Benard instability**. Establishing an increasing vertical temperature gradient (ΔT) across a thin layer of liquid leads to heat transfer through the layer by conduction (organizational state #1). Upon reaching a certain critical value of temperature gradient (ΔT_C_), an organizational state transition takes place within the liquid layer and conduction is replaced by convection (organizational state #2), leading to a stepwise increase in the rate of heat transfer through the layer. The organizational state #2 (i.e., convection) is a more ordered state (higher negative entropy) than the organizational state #1 (i.e., conduction), and, thus, it requires a higher rate of energy/matter flow through the system for its maintenance. The organizational state #2 (convection) will relax into the organizational state #1 (conduction) upon decreasing temperature gradient (not shown). The Benard instability is an example of a nonequilibrium nonliving system displaying a number of the universal (self-) organizational law-like patterns shared by all nonequilibrium system, including living organizations/systems, broadly defined (see discussion in the text). Reproduced from [20].

Since the cell *is *an open nonequilibrium physicochemical system of interacting molecules, the cell is *expected *to exist and function as a complex metastable organization of conjugated fluxes, steady-state compartments, and interdependent gradients. This implies that molecular partitioning, ordering, and macro-organization within the cell are not pre-determined by a pre-existing design, but are driven by the same physical principles and forces that drive self-organization in open, inorganic, far-from-equilibrium systems studied in the field of nonequilibrium thermodynamics. As suggested elsewhere [20], one of the principal differences between nonliving and living organizational processes is that functional constituents of living systems (on each and every level of biological organizational hierarchy) are complex living organizations in themselves, whose structures and dynamics have been shaped (but not specified) by evolution. The structures and dynamics of all living organizations, from proteins and cells to ecologies and societies, embody their evolutionary histories/memories. Therefore, in contrast to inorganic systems, the self-organization of any living organization/system is greatly facilitated, and to a certain degree governed (but not determined), by evolutionary memories embodied in more or less specific, yet flexible and adaptive, structures and dynamics of its interacting constituents. It should be pointed out that, since the organizational dynamics of individual proteins, cells, organs, organisms, and economies display long-range correlations in time [24-29], the structure and dynamics of any living system - be it a protein, a cell, an organism, an organization, or an economy - carry within them the memories of previous experience accumulating at multiple scales. Continuous reproduction of such memories in the form of the specific structures and dynamics of system's constituents (e.g., proteins, cells, organisms, organizations) is that which makes the self-organization and performance of any living nonequilibrium system - be it a cell, an organism, an organization, an ecosystem, or an economy - increasingly fast, reliable, and reproducible as its experience accumulates.

The other empirical (self-) organizational laws of nonequilibrium thermodynamics that are especially helpful in understanding the organizational dynamics of living systems are as follows. Increasing the rate of energy/matter flow through an open nonequilibrium organization/system of interacting components normally leads to the growth of the organization/system in size and complexity. The increase in complexity proceeds through stepwise organizational transitions from states of relatively low order (low negative entropy) to states of relatively high order (high negative entropy) and is accompanied by the formation of multiscale organizational hierarchies. Maintaining a nonequilibrium organization/system at a given level of order and complexity requires a continuous and stable flux of energy/matter through the system. Decreasing the rate of energy/matter flow through an organization/system leads to a stepwise hierarchical relaxation of its organizational structure and a loss of complexity and order, ultimately culminating in the dissolution and death of the organization [20].

One of the practically important predictions that can be immediately made from these empirical laws is that the critical parameters defining the physiological state of any living system, such as the cell or the organism, for example, are *flow rates *of their constituents (nonequilibrium thermodynamics) and not concentrations, as commonly assumed (equilibrium thermodynamics) [20]. Indeed, a momentary survey of research literature confirms this prediction. The measurements performed on over 60 different metabolites in different metabolic pathways show that intracellular metabolite concentrations are homeostatic and do not change significantly upon transitions in the physiological state of the cell, such as, for example, a shift from resting state to a high workload state, while metabolic fluxes through corresponding pathways change dramatically upon such transitions [30]. At the scale of a whole organism, the steady-state level of glucose in the blood is maintained within a remarkably narrow concentration range, whether after a large meal or during fasting, whether at rest or during endurance exercise. The parameter reflecting the physiological state of the organism is not glucose concentration but the rate of glucose flow/circulation. The same is true for oxygen, phosphate, iron, calcium, and many other metabolites circulating with the blood flow.

The cell should thus be pictured as a multiscale system of structured circulation of energy/matter forms in which individual configurations of energy/matter flow are manifested as steady-state sub-cellular structures, compartments, complexes, and macromolecules that make up the cell. Various metastable configurations/patterns of flowing energy/matter continuously compete and cooperate one with another in order to obtain and to ensure stable and accelerating flows of energy/matter passing through them, which they require for their maintenance and growth within the cellular economy they comprise. In a conceptually analogous way, various business, social, and political organizations compete and cooperate one with another in order to obtain and ensure stable and accelerating flows of resources passing through them, which they require for their maintenance and growth within the socio-politico-economic system they form [31,32]. Those organizations that succeed in securing and accelerating the flow of energy/matter through their structures grow in size, order, complexity, and influence. Those organizations that fail to maintain achieved rates of energy/matter flow through their structures either diminish in their relative size, order, complexity, and influence or dissolve. This implies that whenever one observes the emergence, growth, persistence, or increasing influence of a biological organization/structure, one should assume the existence of a relatively stable, rapid, and accelerating flux of energy/matter passing through the biological organization/structure in some form. The converse is also true: behind the deterioration, disorganization, and dissolution of any biological organization/structure there is always a weakening of the energy/matter flux(es) sustaining the organization/structure [20]. Of note, the interpretation of organisms, ecologies, organizations, and economies in the same conceptual terms is an accepted convention in organization theory [31]. In economics, static general equilibrium theory is ridiculed for being in stark and obvious contradiction with the dynamic disequilibrium of economic reality [32,33]. In his recent book, Geerat J. Vermeij, a distinguished paleontologist and evolutionary biologist, concludes that evolution and economics are one, that human institutions are built and function as living organisms, and that both of the latter should be treated in exactly the same terms within a single theoretical framework [34].

In addition to what can be called the *anima ex anima *principle, another fundamental difference between nonliving nonequilibrium systems and living nonequilibrium organizations is that, while nonliving macrostructures emerge and persist only when provided with a flux of energy/matter, living nonequilibrium organizations *strive to live long and prosper*, i.e., to persist as specific dynamic structures and to grow in size, complexity, and influence. As a consequence, at each and every level of the biological organizational hierarchy, living nonequilibrium organizations continuously compete and cooperate one with another as they search for, obtain, transform, and exchange energy/matter forms to ensure a continuous and accelerating flux of energy/matter, which they need both individually (hence competition) and collectively (hence cooperation) for their maintenance and growth in size, complexity, and influence. As time passes, surviving life forms become increasingly interconnected and integrated - both within and across organizational levels and spatiotemporal scales - into a multiscale continuum of structured energy/matter circulation/flow, which favors cooperation and organization over unbridled competition. In this way, proteins and other molecules self-organize into cells, cells self-organize into organisms, organisms self-organize into ecosystems and organizations, and organizations self-organize into economies [29].

To summarize, the self-organization of living systems is driven by economic competition, obeys empirical laws of nonequilibrium thermodynamics, and is facilitated and, thus, accelerated by memories of living experience embodied in the adaptive structures and dynamics of interacting constituents. Because the structures and dynamics (i.e., behaviors) of individual constituents at all levels of organizational hierarchy always remain ambiguous and unspecified, albeit shaped by evolution and experience, the *individual choice *as a force of both innovation and conservation becomes a decisive factor in organizational dynamics, either promoting or inhibiting the survival and success of the developing and adapting whole.

To conclude, in the domain of life, order and reproducibility do not come from design. Instead, they come from what can be called *knowledge *or *intelligence*, a combined and self-organized product of living experience represented by and preserved in the structures and dynamics of interdependent and interconnected living organizations co-evolving on multiple scales of space and time. Evolutionary memories in the form of proteins, cells, organisms, ecosystems, organizations, and economies continuously recall the past by virtue of their own incessant reproduction, adapt to the present by making individual choices and acting upon them, and mold the future by interacting with and molding their environments that, in turn, mold them. In this way, the past, the present, and the future, as well as multiple spatiotemporal scales, converge within one self-organizing process of life. Life is intelligence. Intelligence implies and involves order, reproducibility, continuous change and development, but no design. Life/intelligence is an open-ended, evolving structure-process of energy/matter flow, a stream of consciousness. And we all are a part of it.

## Competing interests

The author declares that they have no competing interests.

## Authors' contributions

AK is the sole author of this paper and is responsible for developing the concepts and for writing and revising the manuscript.
